# Between utopia and precarity: subsistence labour as embodied technology in Indian ecological documentaries

**DOI:** 10.3389/fsoc.2026.1810124

**Published:** 2026-06-05

**Authors:** Ankita Sundriyal

**Affiliations:** School of Social Sciences and Languages, Vellore Institute of Technology, Vellore, Tamil Nadu, India

**Keywords:** documentary film, ecofeminism, gender and labor, neoliberal development, spiritual greenwashing

## Abstract

This paper analyses two Indian documentaries—*Re-Imagining Leadership* (2022) and *Uttarakhand Women End Water Woes* (2021)—to interrogate how the state-sponsored and non-governmental organisation-led discourses of women empowerment and environmental conservation frame women’s ecological labour, particularly in the state of Uttarakhand, India. While the 2022 documentary presents a near-utopian model of women-led rural governance promoting subsistence economies, its narrative subtly tucks away the reliance on state support, frequently hinted at by the women leaders. This complicates its vision of a sustainable-financial independence and the promise of probable autonomy at the centre. The latter, documenting grassroots water revival initiatives by non-governmental aid, similarly highlights women’s labour as an empowering solution for systemic failures and presents it as an embodied technology for guaranteed success. Drawing on Feminist Postcolonial Ecology, Feminist Science and Technology Studies and Postcolonial Development theory, I argue that these narratives aggrandise women’s subsistence practices/labour as ‘embodied technologies’ to sustain ecological viability, hinging on a promise of increased agency at the centre. By contrasting romanticization of women’s care work as ‘empowerment’, the study exposes the tension between celebrating localised women empowerment and the state’s systematic obscuring of structural dependency and land dispossession. The study, therefore, contributes to debates on feminist sociological debates on gender, decolonial sustainability, and the political economy of visual storytelling.

## 1 Introduction

Women of Uttarakhand, the hill state of India, face a major problem today: increased vulnerability in terms of economic growth and independence, as their traditional participation in subsistence activities, such as farming and livestock maintenance, is systematically devalued. Women form a majority of the agricultural workforce and yet, their land access remains precarious at best, with weak tenure security and low to no visibility of labour (Pattnaik et al., 2018, pp. 146, 148; Rao, 2011, pp. 4–5, 11). Although environmental awareness has been a part of Uttarakhand for decades, state policies continue to prioritise tourism and agricultural production over ecological preservation (Sharma and Chaudhry, 1996, pp. 65–67; Thapa, 2024). With the continued erosion of existing subsistence lifestyle, lack of decision-making power and limited financial opportunities and access, women continue to live under great pressure to opt for the commodity production-wage earning model (Maithani et al., 2025, p. 454; Sati and Kumar, 2023, pp. 1–2, 14; Gooch, 2014, pp. 164–165, 171; Mies, 2014a, p. 211). In the case of the *Pahadi* (hill) women (as the farmer), this is achieved and celebrated through introducing the cash economy and gainful employment in industrial workforce through neocolonial, globalising tendencies, such as contract farming, ecotourism labour and microcredit schemes that incorporate women in global supply chains (Wilson, 2011, pp. 318–319, 323). While Maria Mies and Vandana Shiva both talk in detail about the process, one example of understanding it would be through Silvia Federici’s assertion: the World Bank in particular is against subsistence farming which, by default, subjugates women financially through loans and labour contracts (Federici, 2019, p. 20). Other examples would be government reports trying to celebrate this shift by profiling women who have ‘successfully’ entered the commercial workforce (Government of India, 2020, pp. v–viii, 12). Such revelations are alarming because, for the *Pahadi* (hill people), subsistence has been the primary way of life, one they are being taken away from, especially in the last two decades, through land enclosures, infrastructural activities and commercial development. For instance, the 2018 Uttarakhand Land Amendment Act authorises the transfer of agricultural land in certain hill districts for industrial purposes, tourism and energy projects, mentioning a vague condition of such projects providing employment opportunities to natives (Government of Uttarakhand, 2018, pp. 6–8). An entire way of life is thus being devalued, only to be replaced by a new neo-colonialist mindset of economic growth that marginalises the already marginalised. The fabric of the communal lifestyle of the rural Uttarakhand faces a rupture with the replacement of their life model of subsistence and shared commons, a struggle that is evident in Africans that are victims of natural calamities, government manipulation or internal conflicts (Federici, 2019, p. 37). This threat is gradually approaching the rural parts of Uttarakhand, where subsistence living continues to thrive.

In the opening scene of *Re-Imagining Leadership* (2022), *Pahadi* women sing while planting rice, manifesting on screen a vision of Federici’s enchanting commons; yet, the camera cuts before the day ends when the women likely return to their homes, sweat drenched and hands blistered (Black Ticket Films, 2022a). Reducing their hard work to a neoliberal screensaver, ecological conservation documentaries often present a utopia of communal satisfaction, successfully hiding socioeconomic and domestic hindrances (for a qualitative, ethnographic case study, refer to Gururani, 2002, pp. 235–239; Sharma, 2008, pp. 44–45). Vandana Shiva and Silvia Federici, among other ecofeminists, tend to frame leadership in commons as a potential site of resistance: a vision that is also found emphasised in the visual narratives of ecological conservation (Shiva, 1988; Federici, 2019). I argue that the utopian vision found in Indian development documentaries, like *Re-Imagining Leadership* (2022) and *Uttarakhand Women End Water Woes* (2021) aestheticises women’s collective labour as empowerment and their subsistence labour as embodied and governance technology to further the infrastructural and agricultural ends of the state, or the conservationist ends of the non-state organisations (NGOs).

By embodied technology, I mean how the subsistence work performed by women on a daily basis is discursively framed as a naturalised and self-repairing tool in furthering certain interests, such as, development ends. This concept differs from the commodification of labour which focuses on wage relations, analysing how the body itself is an invisible, cost-free infrastructure. By governance technology, I mean how the state and non-state interventions in conservation practices use women’s bodies and labour as technology to govern such subsistence-dependent lands. The former is relevant to how women’s labour is framed while the latter engages in its instrumental use by institutional actors. Further, this utopia neglects any mention of neoliberal dependency, conflicts related to land use and access rights, and as such, enables green grabbing and green colonialism within the context of economy of repair (Fairhead et al., 2012, pp. 241–242). By removing conflicts from the scene, the narratives become perforated sheets of agrarian economies, ripe with opportunities of financialisation of nature in the name of ‘economy with ecology’ (as opposed to the seminal motto ‘Ecology is the permanent economy’ by the environmentalist, Sunderlal Bahuguna during the 1970s Chipko Movement), enchanting the commons leadership on screen. I term this process ‘spiritual greenwashing’, wherein neoliberal development co-opts the vocabulary of sacred, feminine-coded ecologies to prove legitimacy while erasing indigenous knowledge, using structural violence, and suppressing political resistance. I am analysing how these documentaries discursively construct a link between women and nature. I am not endorsing an ontological claim about women’s innate ecological connection. My focus is on representation and co-optation, not on essentialist claims about gender and the environment.

Two documentaries were chosen as case studies. The first one, titled *Re-Imagining Leadership* (2022), is a series of four films by the Black Ticket Films, two of which are set in villages of Uttarakhand where women have taken charge of the rural leadership and governance to ensure a more sustainable future for the natives as well as the biodiversity (Black Ticket Films, 2022a,b). This film was sponsored by The Hunger Project, which is a “global, non-profit, strategic organization committed to the sustainable end of world hunger” (The Hunger Project, 2024). One of the films is set in Pachisi, focusing on the all women led village governance model while the other focuses on the experienced duo, Tara-Basanti, who train women to take roles of leadership and highlight the contribution of women in rural governance. The other titled, *Uttarakhand women end water woes on their own; revive dry water springs* (2021), is a short documentary sponsored by a non-governmental organisation, Down to Earth, on the acute shortage of water as a result of the drying of mountain springs of Indian Himalayas (Down to Earth, 2021). It follows the efforts of the local community, especially the women, as well as Chirag, an NGO, as they pool finances and resources to forge a system of recharging the springs in the village of Lamgara, Almora district of Uttarakhand. Similar women-led spring revival initiatives in Uttarakhand have been documented elsewhere (Raturi et al., 2022). The former documentary clearly purports the ideal of a utopian commons governance where women hold the authority in managing the rural economy, and maintain a participatory style of leadership, while the latter uses women crisis technologists, leveraging their labour as a tool to solve ecological disturbances. Together, these narratives represent the Janus face of development media: as they celebrate the commons and the leadership within, several shortcomings are swept under the rug of the beautiful landscapes portrayed on screen.

Expansive scholarship that links women to the development agenda, where the discourses of empowerment function in a way that the prevalent structural inequalities remain intact. Andrea Cornwall and Althea-Maria Rivas have pointed to the fact that despite the introduction of the terms, ‘gender equality’ and ‘women’s empowerment’ to the international development agenda since the 1980s, the translation of these concepts from their feminist terrain onto other institutions, such as NGOs or government and mixed agencies has compromised their radicalness (Cornwall and Rivas, 2015, pp.8–10). Srilatha Batliwala traces the journey of ‘empowerment’ as a buzzword from the 1970s, focussing on the denigration of the radical concept within state policy (Batliwala, 2007, pp. 557–561). These studies remain critical to understanding women as mobilised subjects of the development narratives within state and NGO-led operations. Within an ecological framework, the unpaid reproductive labour of women as well as the additional ecological work goes unacknowledged, even though it may be celebrated as the basis of such development projects (Kishwar, 1987, pp. 95–96). The burden of ecological labour continues to fall disproportionately on women, who sustain not only households but also, degraded ecological spheres. This has already been traced in ecofeminist studies that link gender and environment (Carson, 1962; d'Eaubonne, 1974; Ortner, 1974; Ruether, 1975; Griffin, 1978; Daly, 1978) and gender, ecology and economy (Merchant, 1980; Shiva, 1988; King, 1995).

Bina Agarwal examines women’s ecological labour in the Indian context, and posits women as both victims and agents of environmental change by introducing class and gender related implications of environmental degradation (Agarwal, 1992, pp. 142–146). Feminist Political Ecology (FPE), however, emerged prominently as a conceptual lens that keeps gender at the forefront of ecological access and responsibilities (Rocheleau et al., 1996, pp. 4–5). FPE undertakes gender as an analytical variable in shaping the knowledge of ecology and environmental struggles related to resource management and land and water access. FPE remains significant to the understanding of gendered knowledges that operate along local ecological axes, especially to examine how the discourses of science and policy systemically isolate women from their knowledge. Further, Silvia Federici’s Marxist-feminist analysis of commons and new enclosures provides the appropriate framework to highlight the commons as an alternate to capitalist monocultures (Federici, 2019, pp. 167–183). Her take on co-optation through this new enclosure system offers the ideal theoretical tool to analyse documentaries like *Re-Imagining Leadership*, which can visually celebrate a governance while suppressing its radicalness.

The ecological and political dynamics of women’s labour on screen can be addressed through Feminist Science and Technology Studies (STS). Judy Wajcman critiques the gendering of technology as masculine, dismissing other forms of traditional or natural labour that are made to look culturally pale in comparison to sophisticated machinery (Wajcman, 2004, pp.12–22). It is pivotal to understand that, what constitutes as technology is a gendered, cultural process. It is as a result of this that the embodied knowledge of *Pahadi* women, such as their understanding of seed cycles, water management and sustainable farming, is rarely acknowledged as expertise. This erasure of expertise can be extended to Donna Haraway’s concept of ‘situated knowledges’ (Haraway, 1988, pp. 575–599). Her theory criticises a view that arises in vacuum, positing that each experience and perspective contributes to framing knowledges. The chosen documentaries, as proposed, take an omniscient view from above, thus creating an illusion that the only thing that matters is the women’s labour and not their situated and gendered knowledge. Feminist STS allows one to examine the films as not mere representations, but as sites of knowledge production that deskill and depoliticise women’s labour. Even though these tools are significant in their own right, they can be systematically integrated to analyse such promotional media.

At their intersection comes a significant research gap, which can be explored to critique development paradigms and examine production of gendered knowledge. These tools can be systematically integrated to critically analyse promotional media, like the ones chosen in this study. Studies on Himalayan ecology seldom take gender in the visual media as a core object of analysis, as do South Asian representations of political ecology. By synthesising these critical frameworks, this paper argues that such documentaries function as visual enclosures by aestheticising the commons, only to empty it of its radical potential. This study moves beyond a mere analysis of media representation to interrogate visual media as a technological tool for neoliberal governance. I am analysing how these documentaries discursively construct a link between women and nature. I am not endorsing an ontological claim about women’s innate ecological connection. My focus is on representation and co-optation, not on essentialist claims about gender and the environment.

## 2 Analytical framework

Women, when placed in the midst of the ecological narrative, face a representational paradox: the more they find themselves represented, the more exploited they are as labour tech. As will be evidenced in the chosen case studies, development media has provided women a platform to showcase themselves as emerging leaders of sustainability and keepers of ecological traditions; however, the way these policies are celebrated often disguises the gradual shift towards a neoliberal enclosure system (Cornwall, 2018, pp. 2–3). By depoliticizing women’s labour as a technology for ecological crisis management, their subsistence work and lifestyle are repackaged into an immaculate conservation narrative. The literature reviewed for this study takes recourse to the theoretical framework of Silvia Federici’s Marxist-Feminist critique of the commons as well as the ecofeminist-postcolonial philosophy/critique of Vandana Shiva and Maria Mies. Furthermore, feminist scholarship on embodied technology has been referred to, in order to examine how the ecological narrative becomes a site to utilise labour-as-technology and promote enclosures.

In *Re-enchanting the Commons*, Federici (2019, pp. 93-94) studies the commons as historically primary to human existence with the following features: shared resources, egalitarian access, decision making without hierarchy and collectively agreed rules. Women become particularly central to this state of ‘commoning’, for they contribute to both the productive and reproductive fabric of the commune. Lately however, the concept of commoning is being institutionalised, i.e., co-opted as ‘new enclosures’ which monetise the concept by making the commons a property of the state, private entities or global institutions. Development media, then, becomes a potent tool to theorise and promote this new enclosure system where collective management disguises the underlying institutional apparatus as well as the shortcomings that have to be managed by women’s leadership. Maria Mies and Vandana Shiva examine the neo-colonial aspect of this enclosure system through an ecofeminist lens (Mies and Shiva, 1993, pp. 52–53, 171). Development, as a West-generated patriarchal construct creates ‘monocultures of the mind’ and colonises the bodies of women, especially those of the Global South. The already prevalent subsistence system has been extensively worked upon, that discusses how the labour that women invest in such a lifestyle is systematically devalued by the scientific development model of the West and the developing countries, under the influence of the West and global financial institutions (Mies and Bennholdt-Thomsen, 2000; Shiva, 1988). This biologically diverse subsistence model is ignored and replaced by a monocultural catching-up development that heavily relies on the invisible labour of women and creates a monoculture that depletes the indigenous knowledge systems—a potential site of resistance.

In order to analyse the subsistence labour of women with the women’s embodied technological labour, it is essential to turn to feminist scholarship on Science and Technology Studies (STS) and embodiment. STS provides a new framework to understand how technology goes beyond digital hardware or software: technology has now become a site of technological production and political struggles. Judy Wajcman in her seminal work, *Technofeminism* states that feminists have begun to treat technology as a “sociotechnical product” that is both determined and utilised by social relations, leading to the conclusion that technology is never neutral, and even gendered to a great extent (Wajcman, 2004, p. 7). The ‘cyborg metaphor’ achieves a dissolution between human and machine, thereby allowing one to understand the importance of labour as an embodied tool that does not predate technology; it is technology itself (Haraway, 1991, p. 151). The documentaries visually construct *Pahadi* women as cyborgian figures, the camera framing their integration with tools and the landscape as perfectly natural and pre-technological and erasing the boundary between body and tool. Further, Donna Haraway’s concept of ‘situated knowledges’ (Haraway, 1988, pp. 575–599) is particularly important to the analysis of the chosen documentaries. Ecological preservation narratives need to acknowledge the peculiar positionality of the women of Uttarakhand rooted in generations of subsistence farming, forest management and water conservation instead of routinely erasing it. In *Re-Imagining Leadership* for instance, the camera adopts an omniscient, aerial view at times that implies a ‘view from nowhere’ while the women’s voices affirm the presence of a successful model. The narrative stages a performance of their innate and natural labour, however, what is never articulated is their situated knowledge of seed cycles, soil health and water management.

This gendering of technology that examines how the body is viewed as a pre-technological tool to be used without situating it within their expertise of ecological knowledge, is a central idea to the study of such documentaries. The focus remains on the masculinised innovation, not on the expertise or their expediency. To the women of Uttarakhand, technology is not a ware one can compute on, rather it is their subsistence lifestyle constructed with the help of their indigenous knowledge passed on to them from their mothers and grandmothers as well as the skill to make-do with limited resources and their communal management (Gururani, 2002, pp. 235–237; Rao, 2011, p. 575; Gooch, 2014, p. 162). This inherited expertise is systematically erased through promotional media that frame women’s labour as natural and pre-dated to technology.

To analyse the chosen case studies, Feminist Critical Discourse Analysis (FCDA) has been employed. FCDA (Lazar, 2007, pp. 141–144) provides a more relevant framework to understand how gender is produced and naturalised in discourses, especially visual media. It moves beyond superficial content analysis: FCDA asks how representations have the ability to construct gendered power relations through examining aspects like omissions and intent. The primary data were two documentaries: *Re-Imagining Leadership* (2022) which was selected for its explicitly utopian depiction of a women-led commons, and *Uttarakhand Women End Water Woes on their Own* (2021), which was selected for its crisis-response narrative to systemic failures of the State. Both the documentaries are available in the public commons and were produced within the last five years as a response to the 2013 and 2021 Uttarakhand floods. Women’s ecological labour and the subsistence lifestyle are presented as a solution to both the documentaries. The analysis was conducted on the basis of the specific themes derived from the theoretical framework of Federici’s lens of commons, Shiva and Mies’ postcolonial-development theories of invisibilisation of labour and monoculture narrative, as well as Wajcman and Haraway’s understanding of embodied technology and situated knowledges. Both the films were viewed to initially understand the overall narrative and then to look for certain themes such as praxis of commons, stylisation of co-optation and enclosure, invisibilisation of women’s labour, imposition of monocultural ideas and representation of women’s labour as technology.

Then, the films were viewed multiple times to conduct a scene-by-scene analysis, focusing on camera angles, duration of shots, lighting and the audio (background, voiceover, etc.) as well as narrative silences. The claims made in the documentaries were checked against secondary sources such as news reports, studies and reports on Uttarakhand to trace any discrepancies and deliberate erasures. However, the limitation of the study, which is solely textual analysis as the premise, is acknowledged and cannot directly map the reception of these films or the lived experiences of the *Pahadi* women. This limitation needs further participatory research to be undertaken. The analytical framework and coding scheme now established, I move on to a close reading of the two documentaries to demonstrate how these codes operate in practice.

## 3 Conceptual analysis

The tone of the chosen case studies differs significantly in the way they approached the subject matter; however, both engage in erasing the sustaining, sustainable potential of commoning as well as the embodied labour of *Pahadi* women.

Cautionary tales of the necessity of ecological conservation are often accompanied by tales of hope and leadership. A prime example is the four-part docuseries, *Re-Imagining Leadership* (two parts of which are set in Uttarakhand), which begins with a central question: What would it take to re-imagine the future? The abstract question is then answered with visual evidence that directs the gaze at women leaders (Black Ticket Films, 2022a,b). The short documentary begins *in medias res*, with women already at ease, managing the rural economy and talking of having displaced old village patriarchs (Black Ticket Films, 2022a). This visual evidence helps set the tone of a hopeful ambition, one that has been achieved to an extent in the documentary’s setting. At 00:43–01:06, an aerial view of the 200-year-old village of Pachisi is followed by blooming pink flowers, accompanied by a voiceover introducing Hema, who became village head at 21. The camera shows her sitting on the ground in a circle of women, speaking informally. The landscape of the ancient village, the flowers in bloom, and Hema’s grounded leadership presents women’s governance as both natural and modern. This closely echoes the enchanting commons of Federici, however the focus on Hema’s leadership swiftly shifts the attention to a model of charismatic authority.

Effectively taking one concrete example of a village called Pachisi, the narrative adopts an inductive method to debunk the myth of “catching-up development” (Mies, 2014b, 55). The development of the state of Uttarakhand, the natives say, lies in the hands of the next generation—specifically women—whose model of ‘good life’ is not one of hyper-individualism but of a communal ambition to see the village grow. The landscapes in the documentary have nothing to do with spanning skyscrapers or booming technology; instead, they showcase a model of co-existence, symbiosis and community, under the guiding, playful leadership of young women leaders. These young leaders were born and brought up in an environment that is deeply sensitive to the needs of the land and believes in managing resources in a sustainable manner.

It is clear that neither time nor urbanisation have a place in this harmonious model of living and that there is no desirable peak of progress to be met. The only goals to be achieved are those of sustainable living and protecting the hills from a “colonial relationship between Man and Nature” (Mies, 2014a, 56). The women in the documentary are portrayed as free of this colonization—they traverse the landscapes and villages with ease, assume roles of leadership (a style that commands only to promote communal and ecological ties), partake in manual labour, supervise rural economies and are active in challenging those who pose a threat to their forests, often using law and social media to their benefit. Possessing an intimate knowledge of natural resources and armed with a commitment to achieve welfare of all, the thematics of the narrative are clear from the very beginning: women at the centre. In the opening sequence, a woman making tea is subsequently seen pouring herself a cup, instead of serving it to her family, and enjoys it in solitude, wondering about the potential change women could bring if they were to be in charge of the Indian democracy.

At 00:34, the camera lingers on the domestic sphere: a woman gazing at the horizon from inside her home, a burning firewood stove, and cowdung being smeared on the floor. These images root women’s authority in the private sphere even as the voiceover states their hopes and ambition of moving on to public leadership. The musings of women visualising themselves in the public realm are filmed from within the private realm, with a highly effective voiceover that adds to the visual narrative. There are no interviewers or interrogations, only women explaining and evaluating their personal situations, and further linking it to their village. From the first frame, the narrative emphasizes the absolute authority of the women over their village, juxtaposing sequences of groups of women both inside and outside their homes At 01:06, Hema plays kabaddi with other women, laughing congenially. The use of a handheld camera and natural light builds an air of intimacy and spontaneity. This depiction of leadership as embodied and egalitarian reinforces the utopian lens of the documentary. However, the emphasis on congeniality also aestheticises a participatory governance that risks downplaying the friction and structural constraints of rural decision-making.

The all-women council is visually presented as vivacious and unified. At 01:33, a string of women walks down the hillside, visually emphasising unity and collective action. At 04:02, women surround a bonfire, laughing, with Hema positioned slightly above everyone else. One woman speaks of ‘getting a sense of power,’ while another describes ‘an opportunity not to be lost’. A hopeful, inspirational background score reinforces the utopian frame. At 04:23, an aerial shot shows women traversing the cultivated landscape as text overlay proclaims that an all-women panchayat has led to ‘ecological, economic and social transformation’. However, a critical analysis reveals that this utopian presentation engages in a significant erasure of the sustainable potential of commoning as well as the embodied labour of Pahadi women. *Re-Imagining Leadership* presents a utopian commons that is right there for the taking: the commons is not a radical alternative to the present governance but a carefully structured, nearly-there model of governance. The women-led commons governance gradually emerges as a mere enactment of a neoliberal enclosure of potential. The critical analysis of the narrative was achieved through three discursive codes: the co-optation of the vocabulary and practice of commons into a management model, a strategic camouflage of state dependency, and the romanticisation of embodied labour as a technological tool devoid of intrinsic knowledge and expertise. The narrative is about achieving a model that has all the visual representation of the ‘re-enchanting commons’ but the perfect model will come only at the cost of an enclosure by the State/Centre.

This State intervention is crucial, but it is barely there in the narrative. For instance, smatterings of subsidies and aid from block officers are casually mentioned but never explored. At 01:54, a woman is shown using a spade to dig, followed by a close-up of hands planting mud and stones to build a dam while Hema’s voiceover explains how she managed to build roads and irrigation canals. This framing presents heavy manual labour, usually the tasks under the jurisdiction of male-dominated Public Works Departments, as almost effortless women’s work. At 03:33, women walk through the wilderness carrying pyres of fire, discussing their installation of solar lamps. While the scene culminates in a shot of the lamps turning on one by one, visually linking women’s labour to technological modernity, the physical toll of this labour is never acknowledged. The women’s bodies become what I term ‘embodied technology’: self-repairing infrastructure that requires no investment, healthcare, or rest Federici’s ‘new enclosure’ comes into full effect here even though it might not be evident in the film—the new commons is dependent on the very structures it promises to bypass, and therefore deprives women as well as the commons from attaining its full, autonomous potential. Moreover, the shots often focus on women’s hands as tools, sowing seeds and smiling away. This aestheticisation of labour, framing it as natural and even joyful, is given a voice too, but what never makes the voiceover is their knowledge of subsistence and sustainability. The soundscape reinforces this aestheticisation. A gentle, uplifting melody plays in the background, but there are no calls between workers, no clinking of tools, no audible breathing that suggests sonic evidence of physical exertionTheir bodies are displayed as biocultural tools but they are never considered experts of knowledge.

In *Uttarakhand Women End Water Woes* (2021), the narrative represents women’s labour as duty, not expertise, for, the visual logic performs the ultimate deception: the process of labour is a necessary prelude to the triumph of a solution. Stopgaps in the ecological conservation fall upon these women to labour through valourising their communal duty and sacrifice. The quest to restore the dried-up springs does not address the reasons they dried up in the first place: commercial deforestation and climate change. The issue is sidelined in favour of the celebration of women’s efforts towards the restoration. At 06:09, an expert speaking is followed by an aerial shot of women working the land, their faces not visible. The camera anonymises them, framing their labour as collective and interchangeable rather than individual and skilled. At 06:54, Neeta, treasurer of the Water Use Committee in Dubroli Village, explains how Chirag (an NGO) promised to bring water. She states that the women shared their problems with the NGO and ‘made it work’, but quickly adds that the women along with the men built the walls to contain runoff from the spring. Her testimony briefly acknowledges women’s agency, but the narrative immediately folds it back into a depoliticised frame where gender-specific burdens are not examined.

At 08:38, another expert speaks while men and women work together. Survey teams move through the village, followed by scenes of village meetings where men sit at the head and women surround them. Neeta shows designs and explains them to other women, but the visual hierarchy of men at the head and women in a circle reinforces traditional gender roles even within a women-led project. At 10:04, the expert discusses how the community linked spring revival to religion, which helped develop practices to keep the water clean. Shots of people ringing bells in a small temple follow. The sacred is invoked as a tool for conservation, but the film does not explore how this spiritual framing might also discipline women’s bodies or foreclose political dissent.

The logic showcases women’s labour as a technological tool as a necessity to ecological conservation. Sequence after sequence of women digging earth, moving heavy stones and shaping their terrain are placed to acknowledge their physical effort, however the imagery is carefully curated to exclude any evidence and repercussions of the toll it has on the beings involved. The embodied reality is purely visceral in nature, presented in the form of a superficial aesthetic. There are hardly any close-ups of blistered hands, strained backs or audible heavy breathing. The camera never focusses on the wrinkled skins or chronic joint pain or exhaustion, only featuring a meticulous erasure of the women’s corporeal materiality. This presents the body as a self-maintaining, sustainable technology that takes away from its vulnerability. The visual moves seamlessly from women’s labour to flowing water, thus creating a frictionless narrative where success can be achieved with a smile. The living and suffering body is transformed into a naturally available technological tool with zero maintenance, reducing the female body to the ultimate despoliation.

This crucial ideological function of body as technology is performed by the erasure of the body’s materiality. The visual progression from human labour to the outcome of flowing water creates a magical translation that promises success without any harm done. No investment, healthcare or rest goes into the making of such human infrastructure on screen. The neoliberal fantasy of a cost-friendly and resilient resource absolves the state and society at large from taking any responsibility for their worker’s current or permanent well-being. Further, the documentary ends on the note of solution to the presented water woes, making the solution and the journey to it a unidimensional one. Instead of this monoculturation of solutions, a complex, ongoing understanding of the challenge of maintenance, sharing and potential (for external intervention) for such projects is not discussed. In both documentaries, there appears to be a low to no governance, one being a collective model while the other only relying on local labour, instead of local governance. In Part 2/4 of *Re-Imagining Leadership*, the focus shifts to the mentorship of Tara and Basanti, two older women who train potential women leaders (Black Ticket Films, 2022b). At 00:32, Tara and Basanti walk on pathways overlooking the village, discussing women contesting in Panchayat elections. Their bodies are framed as embodiments of experienced leadership, shoulders bent but gaze careful and steps measured. At 01:13, an aerial shot shows them walking on a pathway between farmlands. Similarly, at 04:43, Tara and Basanti traverse bridges, rivers, and villages, symbolising the reach of their mentorship. The landscape becomes a backdrop for empowerment, but the actual content of what they teach is limited to an introductory statement about not being able to contest in the elections. Their strategies, negotiations, and political struggles remain unexplained and at most, hinted at.

The documentary presents governance as a set of technical skills that women must learn. At 01:20, Tara and Basanti sit slightly above a group of women, teaching them how to write proposals. The camera cuts to close-ups of women answering questions and trying to understand, their faces earnest, their bodies leaning forward. At 02:09, the frame focuses on a woman holding a pen and working with files. All of this stands in for governance as a technology of paperwork. At 06:22, Tara and Basanti write motivational songs in the kitchen, blending domestic space with political education. Yet, as with the earlier scenes, the actual content of the proposals, the strategies for navigating bureaucracy, and the specific challenges of governance are never detailed. There is a subtle erasure of political friction as well. First, at 03:03, men appear in the background of a frame, present but not participating. Their presence without verbal engagement suggests acceptance, but the film does not explore their opinion on women’s rise to power. Second, at 05:11, the testimonies from erstwhile and current village heads, SHG members, and ward members affirm the success of the women’s leadership. There are no dissenting voices, no accounts of failure, resistance, or conflict. This is a hallmark sign of what I have termed spiritual greenwashing that erases the often messy reality of gender politics in rural Uttarakhand.

*Re-imagining Leadership* is more subtle in its approach to vocabulary of co-optation by hiding the state support and validation, while *Uttarakhand Water Woes* is more explicit in exposing systemic failure but efficiently moves the focus away to aestheticised women’s labour. The former also indulges in a kind of erasure, that of indigenous knowledge systems, replacing it with joyful labour while the latter erases all physical cost and fatigue completely. All of this is achieved through romanticisation: in one case, the body is in harmony with nature while in the other, the body is a technological tool for crisis management. The overall function that both narratives serve is appropriating the concept of commons as well as naturalising women’s labour for a neoliberalist economy. Both the utopian and crisis narratives ultimately contain the radical potential of women’s ecological labour and knowledge, through disembodying their labour and turning it into a manageable, inexhaustible resource.

## 4 Discussion

Both the narratives chosen in the study represent seemingly differing viewpoints on the methods to approach conservation efforts in Uttarakhand. However, ideologically, the narratives are not that different: both have a similar modus operandi that I call *spiritual greenwashing*. By spiritual greenwashing, I mean the process wherein neoliberal development co-opts the vocabulary of sacred, feminine-coded ecologies to prove legitimacy while erasing indigenous knowledge, using structural violence, and suppressing political resistance. Unlike conventional greenwashing, which focuses on corporate deception about environmental practices, spiritual greenwashing weaponises feminine divinity and devotional labour to position ecological degradation as acts of reverence. As Kabeer (1999, p. 2) notes, the translation of feminist concepts into policy discourse often strips them of their radical edge, something that is evident in how these documentaries reframe women’s labour as apolitical ‘empowerment.’ The feminine aura of Nature in the state is leveraged by both state and non-state entities to achieve a palatable version of development that tucks away the material conditions and political struggles of the Pahadi women. *Re-imagining Leadership* and *Uttarakhand Women End Water Woes,* bring the dual framework of utopia and crisis to perform a discursive enclosure of the commons.

In the former, commoning as a social vision is co-opted by these narratives, packaging it as a state-friendly governance model. However, state enabled permissions or state-led interventions and checks remain slightly hinted at, exposing the painful reality of a new enclosure system where the commons inevitably exist at the mercy of the State. In the latter, the non-state entity performs an enclosure that is bodily. It manages to erase the corporeality of the labouring female body, along with its fatigue, and showcases it as a resource and technological tool that can be endlessly exploited. The body becomes a solution for institutional failure and requires no investment. The two documentaries emerge from different institutional contexts. *Re-Imagining Leadership* was produced by Black Ticket Films for The Hunger Project, an international NGO that promotes community-led development. *Uttarakhand Women End Water Woes* was produced by Down to Earth, an Indian environmental journalism outlet. Despite these differences, both deploy similar visual and narrative strategies of spiritual greenwashing. However, the NGO film emphasises replicable ‘models’ of governance, while the journalism piece leans on crisis rhetoric and expert testimony. A fuller analysis would require attending to how institutional mandates shape aesthetic choices.

The strategy adopted by such narratives is that of political-economic erasure. The romanticisation of the commons and the instrumentalisation of women’s labour in these films is not merely an aesthetic choice; it serves a critical function within a longer history of primitive accumulation and contemporary land privatization in the Himalayas. The state way forward with economy with ecology does not undo the fact that a system of ecology was already in operation in the hills, albeit it was based on a local, subsistence agrarian lifestyle. State policies, such as the 2020 Uttarakhand land protection act, restrict the conversion of agricultural land for non-agricultural purposes, yet large-scale infrastructure and tourism projects continue to be exempted (Uttarakhand Investment Promotion Board, 2020). The tendency of primitive accumulation by the capitalist class has throughout the history of the world attempted to destroy communal relations through the enclosure system and acquiring labour (Federici, 2019, p. 87). The privatisation of land today by government and commercial projects is a similar takeover of the land and water use of the *Pahadi*, who have age-old ties with the ecology and live in a state of interdependence.

The logic used for such commercial agriculture rests on the popular myth of ‘feed the poor’. The entire eco-agricultural system that is a part of a commons rests on the creative, regenerative capacity of the seed; it is the protection of this seed that leads the production in a commons-based society. Contrary to popular belief, 70% of the world’s food still comes from local farming communities that rely on ecological diversity (Shiva, 2016, p.11.4). The commercial agricultural practices promoted by capitalist industries that stand behind the government promise mass production to an ever-growing population, blaming the poor half for its population explosion. However, industrial agriculture only promotes monocultures that produce crops guaranteed to make profits, whereas small farms that are inherently cooperative and sustainable actually promote food security (Shiva, 2016, p.11.5). Globalising agriculture means an erosion of these small-scale subsistence-based farming practices that benefit both the farmers and consumers through ensuring livelihood for one and nutritiously diverse food for another. Reducing seeds to mere commodities to be owned by private enterprises and the natural capacity of regeneration to the innovative expertise in laboratories goes against the ethics of the interrelationship between human beings and ecology. Women, ultimately, are framed as custodians of such a system, even though historically speaking, they have been the defenders of Nature against such practices.

This study is limited to textual analysis of the documentaries themselves. It does not measure how these films are received by audiences, nor does it assess their impact on policy or public opinion. However, it is worth noting that both films circulate in specific institutional circuits. *Re-Imagining Leadership* is promoted on The Hunger Project’s website and likely screened at NGO workshops and donor meetings. *Uttarakhand Women End Water Woes* is publicly available on YouTube and has been shared by environmental advocacy networks. Future research could examine how these films are actually used, interpreted, and contested by different audiences. This study aims to contribute to the ongoing concept of ‘embodied technology’, using Feminist STS along with Feminist Political Ecology to understand how women are instrumentalised as tools of technology and labour. Wajcman’s critique of gendered technology and Haraway’s ‘situated knowledges’ highlight the manipulation in the visual economy in such narratives. The spiritual greenwashing changes the political struggle of the *Pahadi* into an organised scrounging for natural resources, and a communal movement into isolated acts of individual resilience. These documentaries, therefore, are not passive narratives but agents in enforcing a depoliticised, neoliberal governance.

## 5 Conclusion

To safeguard the true idea of a communal society from commons in disguise or commons used to merely produce commodities, one can rely on Federici’s definition of commons: Commons should be a community that is autonomous, self-governing, unified in interests and freely share property such as natural or social wealth which includes all natural or man-made resources; commons should be beyond control of the State or the market in order to limit ‘capital accumulation’ (Federici, 2019, pp. 15, 93). A commons, however, may appear to be a tedious, unproductive system where time is built on the concept of finding a common ground for communal development, and not time spent on productive use to make commodities, and hence profits.

The role of women in the idea of commons is both historically and politically significant: historically because women have been at the heart of these communities, protecting the interests of a sustainable, subsistence lifestyle by driving away both illegal and government driven commercial attack on lands and forests (examples being Chipko and Anti-Tehri Dam Andolan movements), and politically, because women remain active in both reproductive and subsistence work in such communities at the grassroots level. As women and Nature both give and sustain life, women have been consciously and unconsciously drawn into the struggle of protecting the ecology, relying on the system of commons for shared responsibility and support. In the state of Uttarakhand, women have been primarily responsible for constructing, maintaining and upholding the idea of a communal, subsistence living that adopts a nurturing stance towards Nature, and while the burden may seem daunting, they are the ones who become the most vulnerable in a state of ecological crisis, whether man-made or natural. Protecting this lifestyle becomes essential to *Pahadi* women if they are to protect their right to life through right to land, water and forest use as well as agricultural production. Built primarily on the idea of a shared living and cooperative decision-making, such a system also prevents the violence of the scientific-led capitalist, patriarchal model that both marginalises the worker and the farmer by taking away their human rights and decision-making.

This paper argues that promotional documentaries orchestrate a critical ideological function through an ecological imaginary by co-opting the concept of commons and the gendered body of the *Pahadi* women. The documentaries are examined through feminist critical discourse analysis to understand their convergence into spiritual greenwashing that aesthetises sacred ecology and women’s ecological labour to serve certain agendas. This study emphasises the combination of macro-political critique (Federici, Shiva) and micro-discursive analysis (feminist STS) to understand the flow of power in the visual economy. It moves beyond celebrating the success of women’s role in ecological conservation and cultural practices and interrogating how that role can be used to instrumentalise subsistence labour for development media. Ethical storytelling ought to move beyond women-as-spectacle and treat them as living creatures with their own struggles, knowledge and demands. Further, an acknowledgement, even if slight, of land rights related conflicts, history of displacement as well as the political economy of the region is required. The gaze of the camera needs to take in not just the smiling faces and gratifying labour of women, but also, the corporations, land acquisition offices and resistance movements.

The protection of the true commons, an autonomous and self-governing community that shares natural resources beyond State and market control, is closely tied to the protection of its narrative. The *Pahadi* community struggles for access to land and water but also for narrative control. The *Devbhoomi* (land of Gods, another popular term for Uttarakhand) needs to emerge from its branded imaginary and navigate through a decolonial, feminist politics to recognise the Pahadi woman as an agent of political ecology, and not just a symbol of sustainability politics. Such co-optation of sacred and feminine ecologies examined in the study is not confined to the state of Uttarakhand. Subsistence communities are framed as spiritual custodians of ‘virginal’ landscapes and their labour is aestheticised. I call this phenomenon ‘spiritual greenwashing’, a concept that warrants further theoretical development. Although my study focuses on two documentaries from Uttarakhand, the concept of ‘spiritual greenwashing’ has a much broader applicability. Future research could examine how sacred and feminised ecologies are co-opted across varied religious and cultural scenarios in other Himalayan regions such as Nepal, Bhutan, Ladakh, and how such communities negotiate and resist the spiritual greenwashing in their ongoing struggles for land and livelihood. It could also explore other forms of development media, including corporate sustainability reports and government tourism campaigns.
